# Melatonin and Mesenchymal Stem Cells as a Key for Functional Integrity for Liver Cancer Treatment

**DOI:** 10.3390/ijms21124521

**Published:** 2020-06-25

**Authors:** Ehab Kotb Elmahallawy, Yasser Mohamed, Walied Abdo, Tokuma Yanai

**Affiliations:** 1 Department for Management of Science and Technology Development, Ton Duc Thang University, Ho Chi Minh, Vietnam; ekelmahallawy@tdtu.edu.vn; 2 Faculty of Pharmacy, Ton Duc Thang University, Ho Chi Minh City, Vietnam; 3 Laboratory of Kafr El Sheikh Fever Hospital, Kafr El Sheikh Fever Hospital, 33511 Kafr El-Sheikh, Egypt; yasser.biology.dept@gmail.com; 4 Department of Zoology, Faculty of Science, Tanta University, Tanta 31527, Egypt; 5 Department of Pathology, Faculty of Veterinary Medicine, Kafrelsheikh University, Kafr El Sheikh Governorate 33516, Egypt; waliedsobhy40@gmail.com; 6 Laboratory of Wildlife and Forensic Pathology/Biomedical Science Examination and Research Center, Department of Veterinary Medicine, Faculty of Veterinary Medicine, Okayama University of Science, Okayama 700-8530, Japan

**Keywords:** melatonin, mesenchymal stem cells, liver cancer, functional integrity

## Abstract

Hepatocellular carcinoma (HCC) is the most common hepatobiliary malignancy with limited therapeutic options. On the other hand, melatonin is an indoleamine that modulates a variety of potential therapeutic effects. In addition to its important role in the regulation of sleep–wake rhythms, several previous studies linked the biologic effects of melatonin to various substantial endocrine, neural, immune and antioxidant functions, among others. Furthermore, the effects of melatonin could be influenced through receptor dependent and receptor independent manner. Among the other numerous physiological and therapeutic effects of melatonin, controlling the survival and differentiation of mesenchymal stem cells (MSCs) has been recently discussed. Given its controversial interaction, several previous reports revealed the therapeutic potential of MSCs in controlling the hepatocellular carcinoma (HCC). Taken together, the intention of the present review is to highlight the effects of melatonin and mesenchymal stem cells as a key for functional integrity for liver cancer treatment. We hope to provide solid piece of information that may be helpful in designing novel drug targets to control HCC.

## 1. Introduction

The last few years have witnessed extraordinary increase in the reports of liver cancers worldwide [1,2]. In 2018, around 841,000 cases and 782,000 deaths were recorded due to these types of cancer [1,2]. Furthermore, this type of cancer has been considered the 5th cancer type between the male, the 7th between female and the 4th fatal between other cancers [1,2]. Therefore, it is not surprising to state that liver cancer has been considered one of the tumors with the fastest rising incidence and highest mortality in recent years [3,4]. Among others, hepatocellular carcinoma (HCC) has been considered the most common type of liver cancers.

HCC is mostly linked to miscellaneous predisposing etiologies including viral hepatitis or exposure to toxins such as aflatoxin [5,6,7]. Given its global distribution, most HCC cases are estimated to occur in Asia and sub-Saharan Africa [6,8]. Several factors are considered inclining agents for developing HCC such as hemochromatosis and alpha 1-antitrypsin deficiency and metabolic syndrome [2]. Taken into account, the prognosis of HCC mainly depends on several factors including the degree of tumor spread, size of tumor and the general healthy status, among others [6]. Despite the great progress achieved in understanding of HCC, resistance of apoptosis treatment is still challenged by apoptosis resistance [9]. Some recent studies showed that inhibitor of apoptosis proteins (IAPs) have been involved in resistance to apoptosis in HCC through inhibition of caspases activation [10,11]. Indeed, it seems mandatory searching effective novel therapeutic agents that can improve the treatment courses of HCC and future prognosis of such cases.

To our knowledge, melatonin has been identified as a natural antioxidant with numerous immunoenhancing properties, while mesenchymal stem cells has shown a potential promising strategy either in preventing or arresting neoplastic growth [12,13]. The intention of the following sections is to give an overview about mesenchymal stem cells (MSCs) and highlight several physiological and biologic effects of melatonin followed by discussing the potential promising effects of the combined use of melatonin and MSCs in treatment of HCC.

## 2. Mesenchymal Stem Cells (MSCs)

### 2.1. An Overview of Mesenchymal Stem Cells (MSCs)

Mesenchymal stem cells (MSCs) are multipotent cells capable for differentiation into cartilage, bone, muscle, tendon, ligament, fat and hepatocyte. MSCs population is one of the major stem cell populations in the adult bone marrow [14]. They represent only 0.01% to 0.001% of all mononuclear cells in the bone marrow [14], making the identification of a native MSC niche is difficult [15]. Several locations for in vivo MSC niche within the bone marrow have been proposed including the periosteal niche, the pericytic niche and the perivascular niche [16]. Surface marker expression studies referred to the perivascular niche as the true “home” for MSCs, allowing the easier access of MScs progeny to the circulation [17]. The recent years gave more attention towards pluripotent mesenchymal stem cells, which are found in bone marrow stem cells (BMSCs) and adipose tissue (AD-MSC) [18]. In this regard, MSCs have been proposed as promising sources for restoring tissue and organ function [19]. However, several potential health hazards for their clinical application were reported, including shortage in their availability, their sensitivity to toxic environments, senescence and tumorigenicity [19]. In addition, MSCs- based treatment has shown regeneration of organ function via the production of cytokines and other several anti-inflammatory mechanisms [20].

### 2.2. Isolation and Characterization of MSCs

To authors’ knowledge, MSCs are mostly isolated from bone barrow, fat and cord tissues [21,22]. However, pluripotent stem cells could be isolated from other numerous tissues and organs, including adipose tissue, skin, dental tissues, placenta, umbilical cord blood, liver, menstrual blood, dental tissue, perinatal tissues and ear [23]. Among others, isolation of MSCs from adipose connective tissue has been considered as an ubiquitous techniques in stem cell-based therapy with minimal invasive protocol [24]. In addition, the outer surface of the ear has plenty of MSCs expressing multiple stromal markers besides their ability to differentiate into different lineage including fat, cartilaginous and osseous tissues [25,26]. It is noteworthy to mention that some modifications have been carried out for isolation of mulitpotent MSCs, including alterations to culture media supplements and serum percentage, growth on various substrates such as collagen and fibronectin, and depletion of hematopoietic cell contaminants by surface marker-based negative selection [27]. These methods allow enrichment of a fibroblastic spindle-cell population [27]. Although a heterogeneous mixture of spindle cells, star-shaped cells and large flattened cells is frequently observed, a characteristic pattern of surface marker co-expression indicates the self-renewal and multipotence capabilities was noticed [28]. Additional surface markers reveal subpopulations of MSC that are differentially committed to various stromal cell types were also recorded [29]. Taking into account that human and murine MSC generally are not able to express hematopoietic markers cluster of differentiations (CD) as CD34 and CD45, however, subpopulations of cells express a low level of these markers [30]. Human MSC were reported to be positive for the following surface markers: CD44, CD73, CD90, CD105, CD106 and STRO-1, while murine MSC express stem cell antigen-1 (Sca-1 or Ly6A) and all these previously mentioned markers except STRO-1 [31]. Likewise, these markers have been used in combination to get pure MSCs from bone marrow isolates [32]. Additionally, it was reported that certain multipotent bone marrow stromal cells did not display these markers in vivo. Consequently, sorted MSCs populations may not contain all multipotent marrow stromal subtypes [33,34]. The absence of MSCs specific genes and markers is another hurdle faced in the characterization of these cells. The trypsin-resistant antigen denoted STRO-1 remains the best choice, since it is absent in peripheral tissues, the hematopoietic compartment or in mature mesenchymal cells, however, it is expressed on endothelial progenitors [35]. Unfortunately, the structural and functional characteristics of STRO-1 have yet to be determined combined with STRO-1 negativity in mice before its use in preclinical studies [36]. It is noteworthy to state that the pluripotent stem cell-derived MSCs overcome many disadvantages of adult MSCs such as reduced batch-to-batch variations and stem cell senescence and produced some unique cytokines different from bone marrow-or cord-derived MSCs [37,38,39]. Interestingly, GMP-grade MSCs are currently being used in clinical trials for various viable diseases, including industrial sectors. In this concern, Mayo Clinic initiated a phase I/II trial to find the side effects and best dose of MSCs infected with oncolytic measles virus encoding NIS (MV-NIS) and to observe its effect on patients with ovarian cancer. These studies suggested further future in-depth research about MSCs timing in patients [40,41].

### 2.3. Recruitment of MSCs

Tissue repair, inflammation and neoplasia represent few of the processes that encourage engraftment of circulating MSCs [42]. Tumor tropism of MSC can be examined by cell trafficking assays in vitro and in vivo. A number of modalities, such as intravenous injection, utilizing fluorescence, magnetic resonance and bioluminescence, can be used to track ex vivo expanded MSC [43]. No previous studies have deeply explored the migration of endogenous MSC into tumors, but some previous reports have shown that recruitment of labeled MSC home to tumor stroma following bone marrow engraftment in sublethally irradiated mice [44].

The process of MSCs recruitment into tumors follows a similar pattern of recruitment of the activated inflammatory cells during tissue repair [45]. MSCs show graded responses to leukocyte and endothelial activating Transforming growth factor-beta (TGFβ-1), interleukin-6 (IL-6), IL-8, IL-37 and neurotrophin 3 (NT-3) [46]. However, under hypoxic conditions, breast cancer cells produce high amounts of IL-6 which activates and attracts MSCs. It should be stressed that IL-6 also acts in a paracrine fashion on MSC, resulting in activation of STAT3 and MAPK signaling pathways that together trigger the survival of the cell and their migratory potential [47]. Furthermore, LL-37 (Leucine leucine-37), which presents in many tumors, stimulates the migratory activity of MSCs and facilitates the progression of ovarian tumor via recruitment of MSCs to act as pro-angiogenic factor-expressing tumor stromal cells [48].

In addition, tumor cells secrete many chemoattractants that promote the migratory activity of MSCs and MSCs express receptors of the four chemokine subfamilies: CC, CXC, CX(3)C and C [49]. It should be born in mind that several chemotaxis assays in vitro have shown that Dose-dependent migration of MSCs could be induced by chemokines like CCL2/MCP1 (monocyte chemoattractant protein-1), CCL25 (thymus expressed chemokine), CXCL8 (IL8), CXCL12/SDF1α and CXCL13 (BCA1) [50]. In addition, sphingosine 1 phosphate (S1P) exerted a strong chemoattraction on MSCs through matrix metalloproteinase (MMP)-mediated signaling events and the RhoA/ROCK and MEK1/ERK intracellular pathways [51].

### 2.4. Dual Roles of MSCs in Liver Cancer

**Figure 1:**
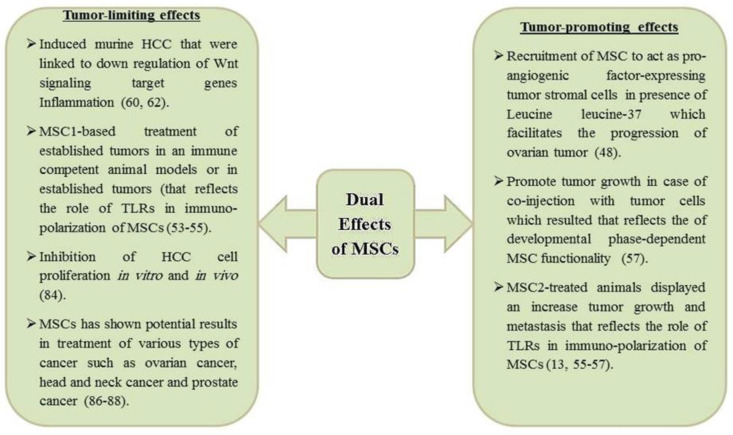
The pleiotropic functionality of mesenchymal stem cells (MSCs) which tumor-limiting and tumor-promoting effects.

It is noteworthy to state that the considerable difference of intrahepatic microenvironment from other organs seems to influence the development of cancer [52]. MSCs constitute an important components within the microenvironment of both normal liver and liver with tumors, suggesting their pleiotropic functionality which is shown in Figure 1. Clearly, MSCs may exert a tumor-promoting or a tumor-limiting effects depending on the experimental circumstances [13]. Several hypotheses have been proposed to explore this dualistic behavior of MSCs in cancer [53,54]. One of these theories L is related to the role played by TLRs in immuno-polarization of MSCs. MSCs express several TLRs combined with their capabilities to migrate, invade and secrete immune modulating factors [13]. Interestingly, TLR4-primed MSCs and TLR3-primed MSCs are polarized into two phenotypes; a proinflammatory MSC1 and the classical immunosuppressive MSC2 phenotype, respectively [55]. In cancer models, MSC1-based treatment of established tumors in an immune competent animal models impaired the tumor growth and metastasis [56]. On the contrary, MSC2-treated animals displayed an increase tumor growth and metastasis [56]. The other theory proposes a developmental phase-dependent MSC functionality [57]. In this hypothesis, MSCs may promote tumor growth in case of co-injection with tumor cells, while their administration in established tumors inhibit progression of tumors. This means that the presence of MSCs during the early stage of tumorigenesis may contribute to angiogenesis [58]. Clearly, the tumor cells and their microenvironment may have an influence on the action of recruited MSCs [59]. Taken together, both postulations seem concomitantly true and therefore, it is very hard to make a prediction to the effects of MSCs on the cancerous process [60,61].

### 2.5. Mechanisms of MSC-Dependent Tumor Suppression in Liver Cancer

To author knowledge, MSCs have shown tumor suppressive effects in induced murine HCC that were linked to down regulation of Wnt signaling target genes [60,62]. There are several suggested mechanisms lying behind this action. In this concern, TLR signals can stimulate downstream effectors that may interfere with LPS–TLR4 pathway and the active secretion of Wnt inhibitors, including Dickkopf-1, combined with MSC-dependent inhibition of NF-kB signaling in cancer cells [13,63]. Additionally, MSCs release microvesicles that exert several actions include inhibition of both cell cycling and the growth of different established tumors in vivo, besides induction of the apoptosis of HCC cell lines in vitro, which in turn provides another antioncogenic pathway [62]. However, it should be borne in mind that the direct effectors are still unclear, but it seems that the secretome of MSCs play an crucial role in suppression of tumors [64]. It should be stressed that various molecular mechanisms seem to be involved in therapeutic MSC actions in vivo and increase their regenerative potential [65]. Among others, direct MSC differentiation into cardiac and endothelial cells and the paracrine activity mediated by MSC-derived soluble molecules and vesicles are currently considered as major factors mediating beneficial effects of MSC-based therapies in various viable diseases [65,66,67,68,69]. However, recent studies showed that paracrine function of MSCs is the main mechanism by which these cells participate in tissue repair [68]. Importantly, MSCs can promote normal tissue regeneration through enhancement of angiogenesis, tissue remodeling and activation of endogenous stem cells [70], which endorsed the paracrine actions rather than cell differentiation. MSCs paracrine functions seem tightly regulated by Rap1/nuclear factor-kappaB (NF-κB) signaling pathway. It is noteworthy to mention that the absence of Rap1 of MSCs markedly enhance the secretion profile of these cells and their resistance to any stressful challenge combined with reduction the production of proinflammatory cytokines [71]. Clearly, NF-kb signaling pathway play a critical role in HCC and therefore, the anti-inflammatory properties of MSCs were found remarkable in the earlier stages [72]. Another interesting mechanism of MSCs is through transfer of mitochondria as reported in several previous studies [73,74]. Mitochondria have been considered a key player involved in many biologic processes in health and disease, including in HCC [75,76,77,78,79]. Some proinflammatory cytokines such as Il-6 and TNF-α can induce MSCs skeletal rearrangement and form tunneling nanotubes (TNT) through which mitochondria mobility occurs from MSCs to neighbor cells [73,80]. Inflammation-driven mitochondrial transfer of MSC to neighbor cells including retinal cells and cancer cells were also reported recently [73]. It should be stressed that the therapeutic effects of MSCs and direction of mitochondrial transfer highly depend on the niche where MSCs is located, including in case of HCC [73]. It seems that the proinflammatory environment can enhance MSCs—mitochondrial transfer and MSC—mitochondrial transfer to T cells, which in turn trigger various immune cells, including CD4^+^T cells [81].

### 2.6. Therapeutic Application of MSCs in Liver Cancer

To our knowledge, several preclinical models showed that MSCs can migrate into different types of tumors and therefore, this notion has inspired many experts in the field about the potential use of MSCs in anticancer drug/gene delivery [82,83]. In this regards, the genetically modified MSCs demonstrated a clear inhibition the proliferation of HCC in vitro and in vivo [84]. Delivering oncolytic viruses such as Measles virus into the tumor cells via MSCs represent another approach to avoid pre-existing immunity against the virus [85]. These interesting findings seems very promising for designing novel trials for treating HCC using MSCs as a vector. In accordance with the clinical trials, MSCs have been extensively investigated in treatment of various types of cancer such as ovarian cancer, head and neck cancer and prostate cancer [86,87,88].

Moreover, adoptive immunotherapy which relies on transfer of naturally occurring or genetically engineered T cells represents another novel shape of cancer therapy [89,90]. This previously mentioned technique could be carried out using induced pluripotent stem cells (iPSCs) that may provide unlimited source of highly reactive antigen-specific cytotoxic T-lymphocytes, which in turn target, infiltrate and eradicate tumors upon their transfer into the patient [91]. Interestingly, bone marrow-derived MSCs transduced with a lentiviral vector stTRAIL have shown promising results in to treatment of heat-shocked residual cancer cells that target tumor growth inhibition [92].

## 3. Melatonin

### 3.1. Synthesis and Precursors of Melatonin

Melatonin (MLT), *N*-acetyl-5-methoxytryptamine, is a natural substance that has been recognized in all major living species including plants, animals, bacteria, other unicellular microorganisms and human being [44,93]. This natural substance is normally secreted during the dark phase of the daily light–dark cycle [94]. Given its lipophilic nature, MLT is mainly produced by the pineal gland, then released into the circulation and gains access to various fluids, tissues and cellular compartments [95,96]. Other peripheral organs and tissues rather than pineal gland are also get involved in secretion of melatonin including retina, Harderian gland, gastrointestinal tract, leukocytes, thymus and bone marrow cells, however, the chronobiotic properties is retained to the pineal secretions [97]. As result of its amphiphilic nature, melatonin gets infiltrated inside subcellular compartment, enabling it to cross all biologic barriers and gets free access to all cellular compartments.

**Figure 2:**
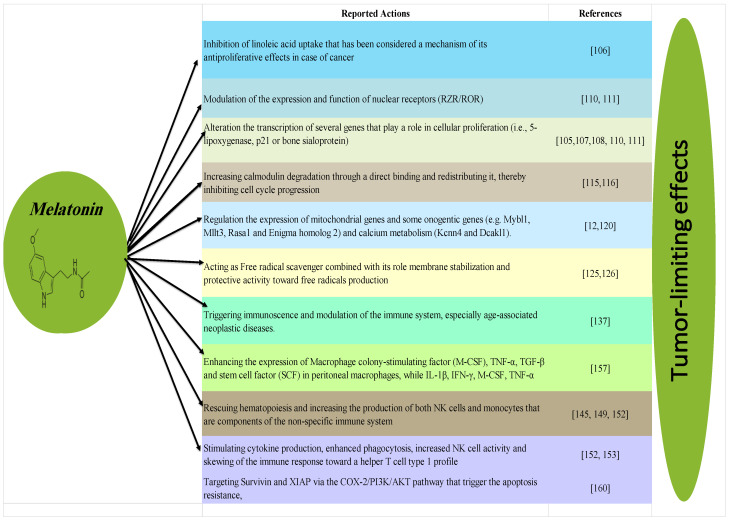
Tumor-limiting effects of melatonin and their suggested mechanisms.

Regarding its synthesis, MLT is synthesized from the amino acid tryptophan, taken up from blood and converted to serotonin [98]. Serotonin is then acetylated to *N*-acetylserotonin by arylalkylamine *N*-acetyltransferase enzyme. *N*-acetylserotonin is subsequently converted into MLT by hydroxyindole-O-methyltransferase (HIOMT) enzyme. Interestingly, the enzymes of MLT biosynthesis have recently been identified in human lymphocytes and therefore, locally synthesized MLT is probably modulate the immune system [99]. Among other extra-pineal major production sites of MLT, the gastrointestinal (GI) tract is of particular interest since it contains several hundred-fold of MLT exceeding those amounts of the pineal gland [100]. Taketo into consideration, GI MLT may release into circulation under certain circumstances such as under the influence of high dietary tryptophan levels [101]. The following section highlights some facts about the physiological and therapeutic implications of melatonin, particularly against cancer (Figure 2).

### 3.2. Signaling Mechanism of Melatonin

To best of author’s knowledge, two mammalian subtypes of G protein-coupled receptor (GPCR) binds to melatonin receptors; MT1 (Mel1a) and MT2 (Mel1b) [102]. These two mammalian subtypes play an important role in exerting some of MLT actions [102]. Moreover, MT3, has been identified initially as third binding site, then it was subsequently characterized as quinone reductase 2 enzyme [103]. It was reported that the decrease in Cyclic adenosine monophosphate (cAMP) production, caused by MLT via MT1 and MT2 receptor interaction, results in reduction the uptake of linoleic acid by affecting special fatty acid transporter [104,105]. Linoleic acid can be oxidized to 13- hydroxyoctadecadienoic acid by 15-lipoxygenase that serves as an energy source for tumor growth and tumor growth-signaling molecules. Moreover, inhibition of linoleic acid uptake by MLT has been considered a mechanism of the antiproliferative effects in case of cancer [106]. Taken into account, MLT also acts through binding to cytoplasmic proteins like the calcium binding proteins such as calmodulin or tubulin and to nuclear receptors like RZR/ROR [105,107,108]. Some studies also suggested that modulation of the expression and function of nuclear receptors (RZR/ROR) could influence the biologic effects of MLT [109,110]. By binding to nuclear receptors, MLT alters the process of transcription of several genes that play a role in cellular proliferation (i.e., 5-lipoxygenase, p21 or bone sialoprotein) [110,111].

Another suggested mechanism of action of melatonin is modulation of intracellular calcium and calmodulin activity [112,113]. Calcium-activated calmodulin is linked to the initiation of the S and M phases of the cell cycle during the cell cycle-related gene expression regulation and in the reentry of quiescent cells from G0 back into the cell cycle [114]. Melatonin has shown to increase calmodulin degradation through a direct binding and redistributing it, thereby inhibiting cell cycle progression [115,116]. It also serves as a potent modulator of gene transcriptional activity and targets a considerable number of genes, in central or in peripheral tissues [117]. It was hypothesized that melatonin mediates the seasonal photoperiodic control via phasing clock genes expression in the pars tuberalis [118]. In addition, MLT downregulates the expression of integrin and integrin-associated protein encoding genes in rat retina, while upregulates the cAMP response element binding protein cAMP response element-binding (CREB) gene in retinal pigmentary cells [119]. Notably, melatonin has also shown a striking effects on the expression of certain genes related to oncogenesis (e.g., *Mybl1, Mllt3, Rasa1* and *Enigma homolog 2*) and calcium metabolism (*Kcnn4* and *Dcakl1*) [12,120]. It should be stressed that the exact mechanisms of melatonin underlying the suppression of these oncogenes are still unclear, however, some reports linked it to the direct interaction of with Bridging Integrator 1 (BIN1) that considers as HCC suppressant gene with c-Myc, leading to downregulation of c-Myc associated with HCC [121]. Interestingly, linked bridging integrator 1 (BIN1) or Myc box-dependent-interacting protein 1 is also highly expressed in pineal gland [122]. In addition, MLT has a significant effects on mitochondrial genes expression, like genes encoding cytochrome C oxidase subunits I and II (*mt-Co1*, *mt-Co3*), 16S ribosomal RNA (*mt-RNr2*), NADH dehydrogenase 1 (*mt-Nd1*),) and ATP synthase subunit 6 (*mt-ATP6*; downregulated) [123].

### 3.3. Antioxidant Effect of Melatonin

Interestingly, it seems that the function of melatonin in phylogeny is related to its antioxidant activity [124]. Several herbs have been used by Chinese in ancient ages due to its high levels of melatonin to retard aging and to treat diseases associated with the production of free radicals [114]. Besides its actions as free radical scavenger and its role in membrane stabilization, melatonin acts on enzymes that generate or metabolize reactive oxygen intermediates, there by further increasing its protective activity toward free radicals [125,126]. Furthermore, melatonin influences the antioxidant enzymes gene expression as it increases mRNA levels for both Cu–Zn–SOD and Mn–SOD in the Harderian gland and brain cortex of rodents [127]. Moreover, melatonin enhances the activity of glutathione peroxidase (GPx) to remove hydrogen peroxide (H_2_O_2_) from cells [128]. Therefore, several important antioxidative enzymes seem to be stimulated by MLT, protecting cells from oxidative damage [129]. Meanwhile, recent report indicated that the engineered MSC with overexpression of GPx can enhance the protection of hepatocytes [130].

### 3.4. Anticancer Effect of Melatonin

To our knowledge, many natural mechanisms are widely known to protect against carcinogenesis, and they fall into two main categories, immune and non-immune [131]. Importantly, immunosurveillance has been proposed as one of the major processes by which cancerous cells are detected and eliminated [132]. Studies of knockout mice have shown the important role played by the immune system in controlling the spontaneous generation of tumors [133]. Understanding the immune changes in the elderly can provide new insights into the complex relationship between immunity and cancer [134]. The age-related impairment of the immune system appears around the sixth decade of age coinciding with a normal decline in plasma MLT concentration [135]. Aging has been associated with a decrease in immune function and an increased incidence of cancer [136]. In this respect, the decline in the production of MLT with aging was suggested to play a crucial role in triggering immunosenescence, especially age-associated neoplastic diseases [137]. antitumor defense assumes a primary role among the various functions attributed to melatonin in modulation of the immune system. The nighttime physiological surge of MLT in blood or extracellular fluid has been proposed to serve as a “natural restraint” for tumor initiation, promotion and/or progressions [138]. The activation of lymphocytes and monocytes/macrophages by MLT can be one of the major mechanisms in preventing tumor development besides its crucial immunomodulatory role in the immunocompromised state [139]. Some previous reports have investigated the potential beneficial effects of melatonin in induction of HCC and HepG-2 cell death) and ovarian cancer in animal models through enhancement the apoptosis of cancerous cells [140,141,142].

Moreover, administration of melatonin increases the production of both NK cells and monocytes (which contain MLT receptors) in bone marrow and spleen within 7–14 days of treatment [143]. Since both cell types are components of the non-specific immune system, melatonin can be effective in prevent the growth of cancer [144]. Indeed, melatonin was able to rescue hematopoiesis from the toxic effect of cancer chemotherapy in several experimental models [145]. This evidence actually poses the basis for the therapeutic use of MLT as an adjuvant in combination with myelotoxic anticancer therapeutic protocols [146].

### 3.5. Regulation Effects of Melatonin on the Immune System

It seems that the level of melatonin secretion in human beings could be influenced along the different season of the year that reflects the significant role played by MLT on immune system modulation [147]. In addition, the synthesis of MLT by human lymphocytes support the hypothesis proposes that MLT has a role in the regulation of immune function [148,149]. Furthermore, melatonin can enhance the immune response that may be helpful in correction of the immunodeficiencies secondary to viral diseases or acute stress [150,151]. It should be stressed that melatonin plays an important role in modulation of hematopoiesis, immune cell production and function [149]. MLT also stimulates cytokine production, enhanced phagocytosis, increased NK cell activity and skewing of the immune response toward a helper T cell type 1 profile (Th1) [152]. Likewise, up regulation of cytokine production and immune function occur as a result of binding of melatonin to its receptors [153]. Both membrane and nuclear receptors have been identified on leukocytes. Importantly, membrane receptors were found mainly on CD4 T lymphocytes, but also on CD8 T and B cells and it was reported that melatonin modulates the proliferative response of stimulated lymphocytes via these receptors [154]. On the other hand, MLT induces cytokine production by mononuclear cells through its influence on the nuclear MLT receptors [155]. Indeed, treatment with MLT enhanced antigen presentation by splenic macrophages to T cells together with a concurrent increase in MHC class II expression and synthesis of the proinflammatory cytokines IL-1, IL-2 and Tumor Necrosis factor (TNF [156].

Additionally, treatment of mice with melatonin resulted in enhancing the expression of Macrophage colony-stimulating factor (M-CSF), TNF-α, TGF-β and stem cell factor (SCF) in peritoneal macrophages, while IL-1β, IFN-γ, M-CSF, TNF-α and SCF was increased in spleen cells of mice [157]. The presence of high levels of melatonin in cultured rat thymocytes and expression of mRNAs encoding for Arylalkylamine N-acetyltransferase (AANAT) and HIOMT in the rat and human thymus cells support the hypothesis that MLT is also synthesized by thymocytes [158]. Likewise, the pineal neurohormone MLT has been widely shown to exert an immunostimulatory and potent inhibitor of apoptosis in immune cells through its action on Th cells and on T- and B-cell precursors, respectively [156,159].

## 4. Potential Beneficial Effect of the Combination between Melatonin and MSCs on Triggering HCC

Given the above information, several previous works have suggested the potential contribution of melatonin to overcome HCC through various mechanisms including targeting the expression survivin and X-linked inhibitor of apoptosis (XIAP) via the cyclooxygenase-2 (COX-2)/phosphatidylinositol 3-kinase pathway (PI3K/Akt) pathway [160]. Furthermore, melatonin has shown to exerts numerous anticancer effects through enhancement the expression of various pro-apoptotic markers (mainly Bax and caspase 3) besides induction of apoptosis and inhibition of oxidative stress, inflammation and angiogenesis [143,161,162,163]. Given the fact that Mel receptors are expressed in bone marrow-derived MSCs, melatonin also exerted various receptor-mediated effects on MSCs including enhancement their survival, motility, engraftment and cell differentiation which seems linked to receptors/matrix enzymes interaction, and therefore higher homing effects of MSCs followed pre-administration of a combination of melatonin with MSCs [163,164,165]. On the other hand, various processes have been postulated in relation to MSC-dependent tumor suppression. In this regard, MSCs pulsed with tumor-derived microvesicles showed an enhanced antitumor activity in HCC [166]. Some previous report revealed the use of melatonin enhanced the potential therapeutic role MSCs in treatment various diseases such as acute kidney injury, metabolic syndromes including diabetes through various mechanisms that include through the activation of antioxidative pathways, inhibition of the inflammatory response and reduction of apoptosis and fibrosis [19,167,168,169,170]. More interestingly, few recent reports revealed the beneficial effects of the combination between melatonin and MSCs on targeting inflammation in HCC [171,172,173,174,175]. However, it should be stressed that there is a clear shortage in the available data about the combined use of melatonin and MSCs and their possible synergistic effects in treatment of HCC. In this concern, some of these reports favor the administration of melatonin before MSCs transplantation and it seems this method offer many advantages over the single use of either factor [172]. Interestingly, pre-administration of melatonin prior to MSCs transplantation in HCC resulted in series of actions include, promoted the homing potential of bone marrow-derived MSCs (BMMSCs) and decrease the carcinogenic effect induced by diethylnitrosamine (DEN) which is known as a potent liver carcinogen in rats [176]. Moreover, a significant decrease in the following parameters has been reported, proliferating cell nuclear antigen (PCNA) index, glutathione S-transferase placental positive foci (GST-P), tumor biomarkers in serum besides reduction of inflammation, angiogenesis and metastasis in HCC [171,172,176]. On the other hand, this combination resulted in induction of apoptosis and antioxidant enzymes, tissue matrix and liver repairs in HCC [171]. This was evidenced by lower level of apoptosis in liver tissues which indicated by marked increase in levels of PCNA immunoreactivity and decrease in levels of fragmented DNA and expression of p53, caspase 9 and caspase 3 genes [171,172,176]. Similarly, another study was carried out by Basyony et al. (2019) revealed that treatment using a combination of melatonin and MSCs were reported to decrease malondialdehyde (MDA) which is a known marker of oxidative stress and the antioxidant status in cancer [176]. In addition, in the same study, this combination increases the superoxide dismutase (SOD), catalase (CAT) and glutathione peroxidase (GPX) combined with attenuation of PCNA, Bcl2 and programmed death ligand 1 (PD-L1) immunostain markers and down regulate the expression of inflammation and cell proliferation genes [171,176].

**Figure 3:**
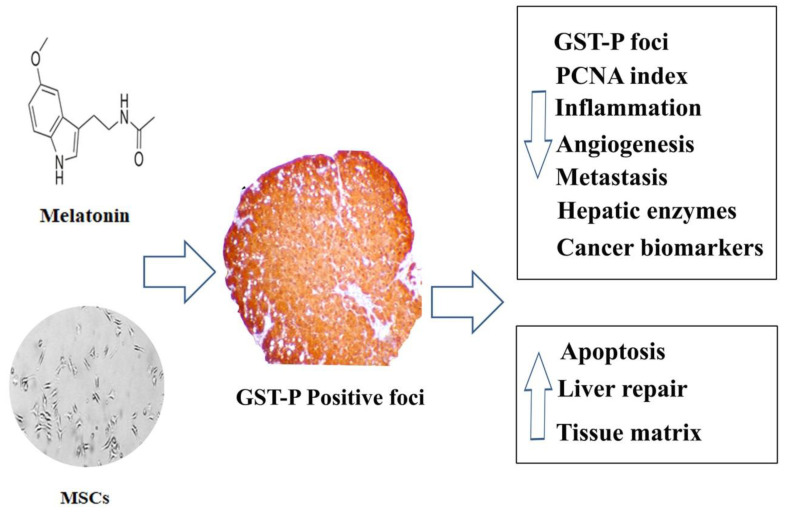
Effects of the combined use of melatonin and MSCs on hepatocellular carcinoma (HCC); ((GSTPS: glutathione S-transferases), (PCNA: proliferating cell nuclear antigen)) [171,172,173,174,175,176].

Taken together, the combined use of melatonin and MSCs may provide promising beneficial effects via triggering the apoptosis resistance and as consequences target HCC Figure 3. It should be stressed that this action was confirmed in rat models where the combined treatment restored the liver function and decreased the HCC versus the treatment with either factor alone or in combination with preconditioning in HCC rats [171,172,176]. Given the lack of available data in this topic, further research seems mandatory to explore more about the effects of this combination together with investigation the main mechanisms underlying the reported actions for better understanding the potential use of targeted stems cell therapy in treatment of HCC.

## 5. Conclusions

In conclusion, the present review highlights the main biologic, physiological and therapeutic effects of melatonin that could be very beneficial for controlling of HCC. Furthermore, an overview about MSCs was given with explaining their potential roles in controlling of HCC and their recruitments and suggested actions. It should be stressed that there is a shortage in the available data that explore the effects of the combined use of melatonin and MSCs-based treatments. The present review suggests further future research for exploring the possible beneficial effects of the combination of MSCs and melatonin in treatment of HCC, together with exploring the role of receptors in this possible underlying activity. We hope that this information may contribute to develop novel drug targets with anticancer activity.

## Abbreviations

AKT	protein kinase B mybl1 MYB proto-oncogene like 1
cAMP	cyclic adenosine monophosphate NF-kB nuclear factor kappa B
COX-2	cyclooxygenase-2 NK natural killer cells
IFN-γ	interferon gamma PI3 K phosphoinositide 3-kinase
IL-1β	interleukin 1 beta rasa1 ras GTPase activating protein
Kcnn4	potassium calcium-activated channel subfamily N member 4 ROR related orphan receptor
MAPK A	mitogen-activated protein kinase member 4 TLR Toll-like receptors
Mllt3	protein AF-9 STAT3 Signal transducer and activator of transcription 3
mt-Co1	mitochondrially encoded cytochrome c oxidase I TGF-β Transforming growth factor beta
mt-Co3	mitochondrially encoded cytochrome c oxidase 3 TNF-α tumor necrosis factor-alpha
mt-Nd1	mitochondrially NADH-ubiquinone oxidoreductase chain 1
mt-RNr2	mitochondrially encoded 16S RNA TRAIL TNF-related apoptosis-inducing ligand
XIAP	X-linked inhibitor of apoptosis protein
